# Down-regulation of mechanisms involved in cell transport and maintenance of mucosal integrity in pigs infected with *Lawsonia intracellularis*

**DOI:** 10.1186/1297-9716-45-55

**Published:** 2014-05-20

**Authors:** Sionagh H Smith, Alison D Wilson, Imke Van Ettinger, Neil MacIntyre, Alan L Archibald, Tahar Ait-Ali

**Affiliations:** 1 Royal (Dick) School of Veterinary Studies, University of Edinburgh, Easter Bush Campus, Roslin, Midlothian EH25 9RG, UK; 2 The Roslin Institute, University of Edinburgh, Easter Bush Campus, Roslin, Midlothian EH25 9RG, UK

## Abstract

*Lawsonia intracellularis* is an obligate intracellular bacterium, responsible for the disease complex known as proliferative enteropathy (PE). *L. intracellularis* is associated with intestinal crypt epithelial cell proliferation but the mechanisms responsible are yet to be defined. Microarray analysis was used to investigate the host-pathogen interaction in experimentally infected pigs to identify pathways that may be involved. Ileal samples originating from twenty-eight weaner pigs experimentally challenged with a pure culture of *L. intracellularis* (strain LR189/5/83) were subjected to microarray analysis. Microarray transcriptional signatures were validated using immunohistochemistry and quantitative real time PCR of selected genes at various time points post challenge. At peak of infection (14 days post challenge) 86% of altered transcripts were down regulated, particularly those involved in maintenance of mucosal integrity and regulation of cell transport. Among the up-regulated transcripts, CD163 and CDK1 were novel findings and considered to be important, due to their respective roles in innate immunity and cellular proliferation. Overall, targeted cellular mechanisms included those that are important in epithelial restitution, migration and protection; maintenance of stable inter-epithelial cell relationships; cell transport of nutrients and electrolytes; innate immunity; and cell cycle.

## Introduction

*Lawsonia intracellularis* is an obligate intracellular, Gram negative bacterial pathogen that causes the disease complex known as proliferative enteropathy (PE). In terms of economic impact this disease is primarily a problem for the pig industry but it also occurs in several other species, though much more sporadically [1-4]. In weaner and grower pigs, clinical signs consist of non-haemorrhagic diarrhoea, ill-thrift and reduced growth rates but in older pigs the consequences of infection can include fulminant intestinal haemorrhage and death [5,6]. In severe cases, PE is characterised by macroscopically obvious thickening of the intestinal mucosal lining which is due to proliferation of epithelial cells which line intestinal crypts, mainly in the distal ileum, though not restricted to it [7,8]. There is a consistent close association between this proliferation and the intracytoplasmic presence of *L. intracellularis* in the hyperplastic crypt epithelial cells [9].

Koch’s postulates were proven in the 1990s and subsequent research has pursued diverse paths, ranging from the development of molecular-based diagnostic tests to the evaluation of various control and treatment options [10-14]. Despite this progress, important mechanistic questions remain unanswered, including the cause of the pathognomonic proliferative lesion and why there are such distinct clinical manifestations of the disease in two different age-groups. There has been limited exploration of the host/pathogen interaction at the cellular level, probably due to the fact that *L. intracellularis* has such rigorous growth requirements [15,16]. The relationship between *L. intracellularis* and the associated crypt proliferation seems to be unique and its potential value as a model for studying the cell cycle, and even the molecular pathways that ultimately lead to cancer, has been recognised for some time [17,18]. Initial analyses of host transcriptional responses to infection using microarrays or RNA-seq support the theory that cell cycle proteins and growth factors are altered in cells infected with *L. intracellularis*[16,19,20]. A number of bacterial pathogens have been associated with cellular proliferation and have been discussed in this context previously [16,21]. One of the main goals of the study reported here was to identify changes in gene expression that might shed some light on host-pathogen interactions occurring in PE. The particular strengths of this work were the analysis of transcript alterations in pigs at several time points following experimental infection with a known strain of cultured *L. intracellularis*, using a comprehensive microarray platform capable of monitoring 47 000 porcine transcripts.

## Materials and methods

### Intestinal tissues

Samples of frozen ileum originated from a previous challenge study [22]. Briefly, 28 seven-week-old pigs were randomly selected from a minimal-disease herd, penned in seven groups of four pigs each and tested for various enteric pathogens. Faecal samples from all animals were culture negative for *Brachyspira hyodysenteriae*, *B. pilosicoli*, *Yersinia* spp. and *Salmonella* spp., and PCR negative for *L. intracellularis*[22,23]. All pigs were also serologically negative for *L. intracellularis*. The pigs were challenged orally with a pure culture of *L. intracellularis* (isolate LR189/5/83), euthanased and subjected to a full necropsy at 3, 7, 14, 21, 28, 35 or 42 days post challenge (dpc), with four pigs per time point. A full-thickness sample of ileum was collected from each pig, snap frozen at -95 °C using isopentane and dry ice, fixed to a cork disk with optimal cutting temperature compound and stored at -80 °C. The results have been described fully by MacIntyre et al. [22]. For the current study a separate group of three uninfected age-matched pigs was used as controls to provide base line data for the gene expression analyses.

### Extraction of total genomic DNA from ileum

Total genomic DNA was extracted from ~100 mg of ileum from 24 pigs using a standard salt extraction method incorporating proteinase K. The quantity of each DNA sample was assessed using a Nanodrop spectrophotometer (NanoDrop Technologies Inc, Wilmington, DE, USA) and the quality was assessed using agarose gel electrophoresis.

### Quantification of *L. intracellularis*-specific genomic DNA

In order to allow measurement of *L. intracellularis*-specific genomic DNA in the total genomic DNA samples, a standard curve for quantitative real time PCR (qPCR) was constructed using a pGEM®-T Vector plasmid which contained a 322 bp *L. intracellularis* ribosomal 16S rRNA gene insert [24]. Serial dilutions of the amplified plasmid were analysed by qPCR using the following primers as previously described [25]: Forward primer, 5′-GCGCGCGTAGGTGGTTA-3′; reverse primer, 5′-GCCACCCTCTCCGATACTCA-3′ and platinum SYBR Green PCR SuperMix UDG (Invitrogen, Paisley, UK). The thermocycling profile used on a Stratagene Mx3000 was as follows: 50 °C for 2 min, 95 °C for 2 min, 40 cycles of 95 °C for 15 s and 60 °C for 30 s. The profile of the final cycle was 95 °C for 1 min, 60 °C for 30 s and 95 °C for 15 s. The standard curve was used to estimate the *L. intracellularis*-specific genomic DNA concentration using DNA of sufficient quantity and quality from the ileal samples (24 of the 28 pigs) and the concentration was expressed as number of 16S rRNA copies per ng of DNA.

### RNA extraction

Total RNA was extracted from the ileum of 20 pigs using Trizol (Invitrogen, Paisley, UK) according to standard methods [26]. The RNA was cleaned using the Qiagen RNeasy minikit following the manufacturers’ instructions (Qiagen, Crawley, UK). RNA was eluted from the spin column in 30 μL of RNase-free water and stored as aliquots at -80 °C. The quantity and quality of RNA were assessed using a Nanodrop spectrophotometer (NanoDrop Technologies Inc, Wilmington, DE, USA) and Agilent 2100 bioanalyser (Agilent Technologies, Palo Alto, CA, USA).

### Quantitative real time PCR validation

The differential expression of several selected genes, as identified from the microarray data, was verified at various time points using qPCR. Reverse transcription was performed as described previously [26,27]. Briefly, one microgram of total RNA was reverse transcribed using a TaqMan kit (Applied Biosystems, Foster City, CA, USA). For qPCR, Platinum SYBR Green PCR SuperMix UDG was used, as described above. The qPCR was performed with a Stratagene MX3000P (Stratagene, La Jolla, CA, USA). Samples were tested in triplicate, GAPDH served as the housekeeping gene and results were calculated as described previously [26,27]. Primers used are listed in Additional file 1.

### Microarray platform and data analysis

To assess if host transcriptional responses were affected, the *L. intracellularis*-infected ileal tissues described above (3, 7, 14, 21, 28 and 42 dpc) were analysed using the Affymetrix Snowball GeneChip® [28]. Sense-strand cDNA was generated from total RNA (500 ng) and subjected to two rounds of amplification (Ambion® WT Expression Kit). The resulting cDNA was used for biotin labelling and fragmentation according to the Affymetrix GeneChip® WT Terminal Labelling and Hybridization protocol (Affymetrix UK, High Wycombe). Biotin-labelled fragments of cDNA (5.5 μg) were hybridized to Affymetrix SNOWBALL arrays using the Affymetrix HybWashStain kit and manufacturer’s recommendations. After hybridization, the arrays were washed and stained using the Affymetrix Fluidics Station 450 and then scanned in an Affymetrix 7G scanner. Image generation and the resulting CEL files for analysis were produced in AGCC – Affymetrix GeneChip Command Console Software. Initial QCs were performed in Expression Console. All microarray data used in the analyses herein are freely available from the Array Express repository under the accession number E-MTAB-1396 [29]. The Affymetrix.CEL files were imported into the Genomics Suite software package version 6.13.0213 (Partek software, Partek Inc.) for data analysis. Transcriptional responses were normalised to those from age-matched uninfected pig ileum prior to running an ANOVA analysis of the data. Up-regulated and down-regulated differentially expressed transcripts at each dpc were selected for further consideration if the false discovery rate (FDR) was ≤ 0.1.

### Gene ontology and pathway analysis

Gene ontology and pathway analysis were carried out using DAVID bioinformatics resources and Ingenuity pathway analysis, respectively [30,31]. Gene expression data were obtained using BioGPS [32].

### CD163 immunohistochemistry

Sections of formalin fixed ileum from all four pigs euthanased at 14 dpc, three pigs euthanased at 42 dpc and one uninfected control pig were stained immunohistochemically to detect CD163 antigen expression. Briefly, after antigen retrieval with proteinase K (Dako UK Ltd., Ely, UK) for 10 min at room temperature, endogenous peroxidase activity was blocked using a commercial blocking agent for 10 min (REAL™ peroxidase blocking agent, Dako UK Ltd., Ely, UK S202386). Following serial washes, sections were incubated with mouse-anti-pig CD163 monoclonal antibody (Serotec Ab MCA2311) diluted 1:30 in Tris buffered saline Tween at pH 7.5. They were incubated with labelled polymer for 40 min at room temperature (Envision mouse HRP reagent, Dako K4001), treated with 3, 3′-diaminobenzidine and counterstained with haematoxylin. Unrelated porcine ileum containing a known macrophage population as defined by a previous study served as a positive control [22]. For each section, the density of immunopositive cells was calculated using image analysis (Olympus Soft Imaging System, Münster, Germany). For each of six randomly selected high power fields (400×) a field of interest was outlined, consisting only of lamina propria, and its area calculated. The number of CD163-positive cells within that specified area was counted per high power field. An aggregate density was calculated for each pig by dividing the total number of CD163 positive cells by the total area examined. A Kruskal-Wallis test was used to compare the aggregate densities for the pigs in the 14 dpc group with those in the 42 dpc group. Assessment of these particular time points allowed comparison of the density of CD163 positive cells at peak infection with the density when infection had virtually resolved. The CD163-positive cell density at 14 dpc was also tested for evidence of a direct correlation with infection burden, as measured by qPCR of *L. intracellularis* genomic DNA. This time point was selected as it corresponded with the highest fold alteration in CD163 gene expression in the microarray analysis.

## Results

### Quantification of *L. intracellularis* bacterial load

**Figure 1:**
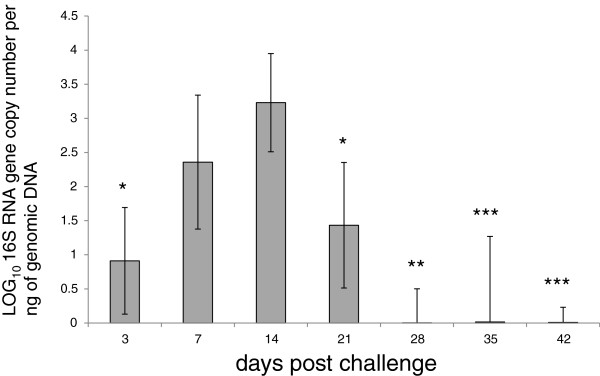
**Quantification of 16S rRNA *Lawsonia intracellularis *LR187/5/83 in experimentally challenged pigs.** 16S rRNA gene copy number per ng of genomic DNA was measured by qPCR as described in Materials and methods. The mean copy number of the 16S rRNA gene was expressed in Log10 for each time point assessed at 3 (*n* = 3), 7 (*n* = 4), 14 (*n* = 4), 21 (*n* = 4), 28 (*n* = 4), 35 (*n* = 2) and 42 (*n* = 3) days post challenge. For age-matched negative controls, 16S rRNA gene was not detected (data not shown). Mean ± standard error are shown. Two-tailed student *t*-test is shown for the most significant comparisons with 14 dpc: *, *P* < 0.02; **, *P* < 0.006 and ***, *P* < 0.0009.

*L. intracellularis* bacterial load was measured in the ileum of infected pigs at 3, 7, 14, 21, 28, 35 and 42 dpc using a qPCR assay of the 16S rRNA gene. The sensitivity of the qPCR test approximated to log_10_ (0.06) copies per ng of DNA. Figure 1 illustrates the pattern of 16S rRNA gene accumulation, with a peak in mean burden at 14 dpc, falling to undetectable levels at 28 dpc. With the exception of 7 dpc the difference between the infection burden at 14 dpc and other time points were statistically significant.

### Host transcript regulation during *L. intracellularis* infection

The integrity numbers for the RNA samples used in the microarray were above 6. In the microarray analysis, the number of transcripts which changed significantly (*p* < 0.1) from uninfected levels was the greatest at 14 dpc (Table 1 and Additional file 2). At all the time points, the number of transcripts that were down-regulated was greater than those that were up-regulated, with peak fold changes broadly occurring at 14 dpc.

**Table 1:** Summary of transcript changes at various time points post challenge (pc)

**Time pc (days)**	**Number of up-regulated genes**	**Number of down-regulated genes**	**Total number of transcripts changed**
3	0	1	1
7	1	3	4
14	26	159	185
21	6	16	22
28	19	18	37
42	2	3	5

FDR < 0.1.

### Validation of differentially regulated transcripts using quantitative real time PCR

Seven differentially regulated transcripts at 14 dpc (fold change ≥ 2.0, FDR ≤ 0.1) were validated by qPCR analysis of RNA samples collected at 3, 7, 14, 21, 28 and 42 dpc. The selected genes were *SLC7A9*, *S100G*, *SLC6A4*, *SLC13A1*, *MUC2*, *TGFBR1* and *CD163*. The coefficient of correlation (R^2^) for each of these time points was produced by plotting the relative level of gene expression as assessed by microarray against the transcript levels generated by qPCR (Table 2, Additional files 2 and 3). With the exception of data at 7 dpc, the microarray and qPCR results appeared to be positively correlated (Table 2).

**Table 2:** Comparison between microarray and quantitative real time PCR assays

Days post infection	3	7	14	21	28	42
R^2^	0.85	0.48	0.91	0.83	0.94	0.87

Seven regulated transcripts with a FDR < 0.05 were randomly selected at 14 dpi. Each primer pair was run with each RNA sample at 3, 7, 14, 21, 28 and 42 dpi. The square of the sample correlation coefficient (R^2^) between the fold changes observed in the qPCR assays and microarray data sets is shown (Additional file 3). Beta-actin transcript levels were used for data normalisation and RNA levels in mock infected ileum tissues were used for normalisation (see in Materials and methods).

### Gene ontology (GO) and Ingenuity pathway analysis (IPA)

Transcripts differentially regulated in *L. intracellularis*-infected enterocytes were analysed with respect to their biological and molecular functions based on the gene ontology data available on DAVID bioinformatics resources (Table 3). Only down-regulated transcripts at 14 dpc showed significant fold functional enrichment as follows: solute/cation symporter activity (9, FDR (false discovery rate) < 2E-02), digestion (16, FDR < 8.01E-06), intestinal absorption (37, FDR < 4E-02) and brush border membrane (27, FDR < 6.3E-04) (Additional file 4). Our results suggest that four broad functional categories were influenced by the presence of *L. intracellularis*. These were cellular transport; brush border/mucosal integrity; local immunity/repair; and cell cycle. Down-regulated genes involved in cell transport included several solute carrier family members (*SLC7A9*, *SLC26A3*, *SLC15A1*, *SLC13A1* and *SLC6A4*), *CUBN* (encoding cubilin, a transporter of cobalamin) and *S100G*, a gene encoding a calcium transporter, which was down-regulated more than 22-fold at time of peak infection (14 dpc) compared to uninfected pigs. Down-regulated genes involved in brush border and mucosal integrity included *CUBN* (suggesting a dual role for this gene), *MUC2*, *RETNLB*, two members of the trefoil factor family (*TFF2* and *TFF3*) and several members of the solute carrier family. Some gene expression alterations indicated that there was suppression of local immunity, specifically a 2- to 3-fold down-regulation of several T-cell receptor complex genes (CD3 delta, epsilon and gamma molecules). In contrast, the greatest fold change in the up-regulated genes was observed in *CD163*, which encodes a protein expressed by scavenger macrophages [33]. We also recorded a 3-fold up-regulation in *SLA-3* (MHC class I antigen) and, finally, the up- and down-regulation of *CDK1* and *HEPACAM2* transcript levels, respectively, both of which influence cellular proliferation.

**Table 3:** Altered cellular networks at 14 dpc identified using ingenuity pathway analysis

**Network functions**	**Score**	**Focus molecules**
Cancer, Gastrointestinal Disease, Cellular Function & Maintenance	53	25
Lipid Metabolism, Molecular Transport, Small Molecule Biochemistry	44	22
Cellular Function & Maintenance, Developmental Disorder, Endocrine System Disorders	28	16
Cell Morphology, Organ Morphology, Molecular Transport	27	15
Endocrine System Disorders, Reproductive System Disease, Developmental Disorder	24	14
Energy Production, Lipid Metabolism, Small Molecule Biochemistry	20	12
Gastrointestinal Disease, Inflammatory Disease, Inflammatory Response	20	12
Cell-To-Cell Signalling and Interaction, Cell Signalling, Behaviour	10	7

Eight cellular networks most frequently associated with altered gene expression in *L. intracellularis* infected pig ileum. As described by [31], the network function score ranks the network and takes into account the number of Network Eligible molecules in the network and its size, as well as the total number of Network Eligible molecules analysed and the total number of molecules in the Ingenuity Knowledge Base that could potentially be included in networks.

### CD163 regulation during *L. intracellularis* infection

**Figure 2:**
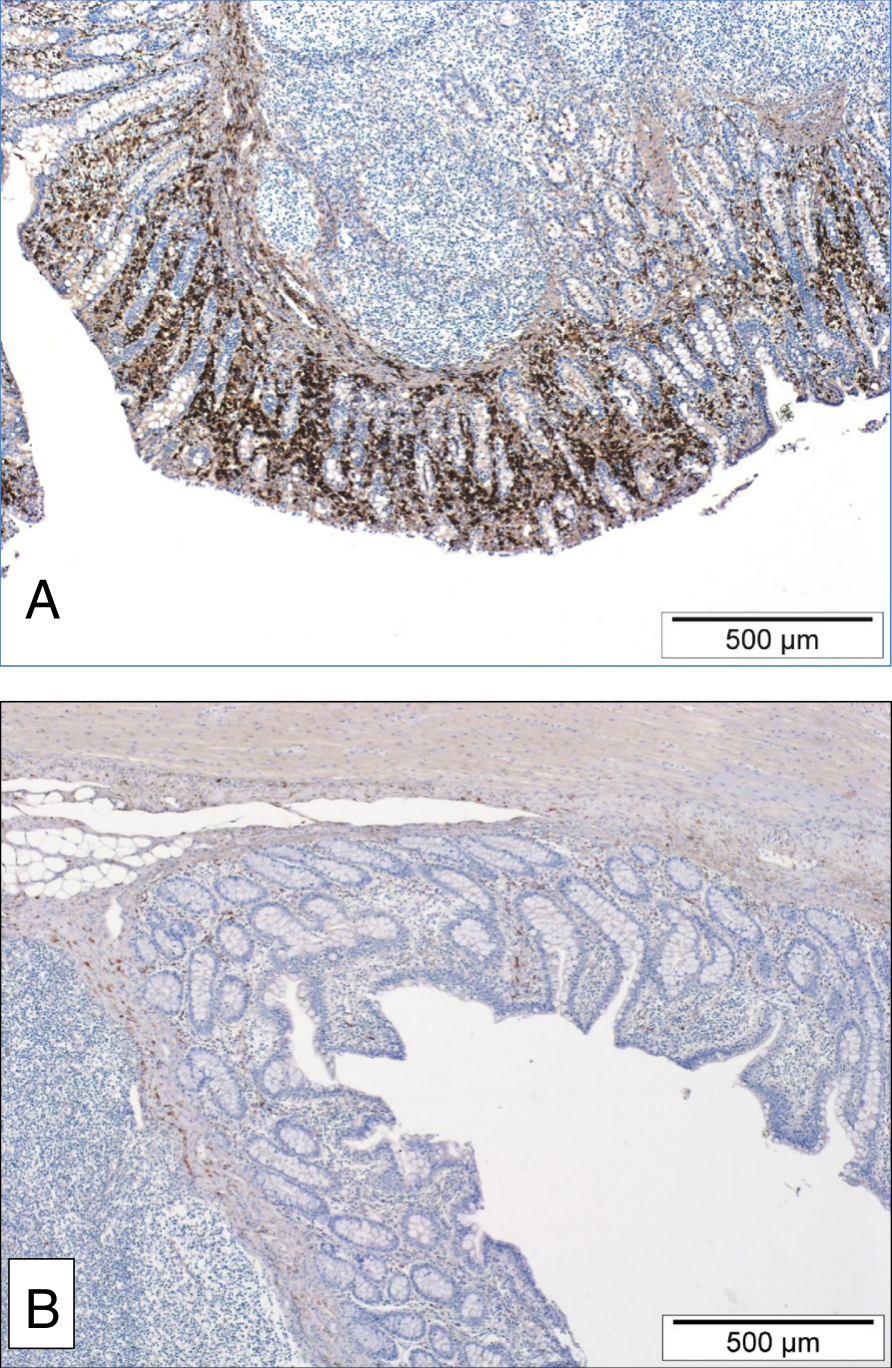
**Pig ileum 14 days post challenge (A) and 42 days post challenge (B).** Large numbers of CD163-expressing macrophages diffusely infiltrate the lamina propria of ileum collected from a pig at 14 dpc **(A)**. This is in contrast to the much smaller numbers of CD163-expressing macrophages in the lamina propria of ileum collected from a pig at 42 dpc **(B)**. Envision HRP and haematoxylin counterstain.

**Figure 3:**
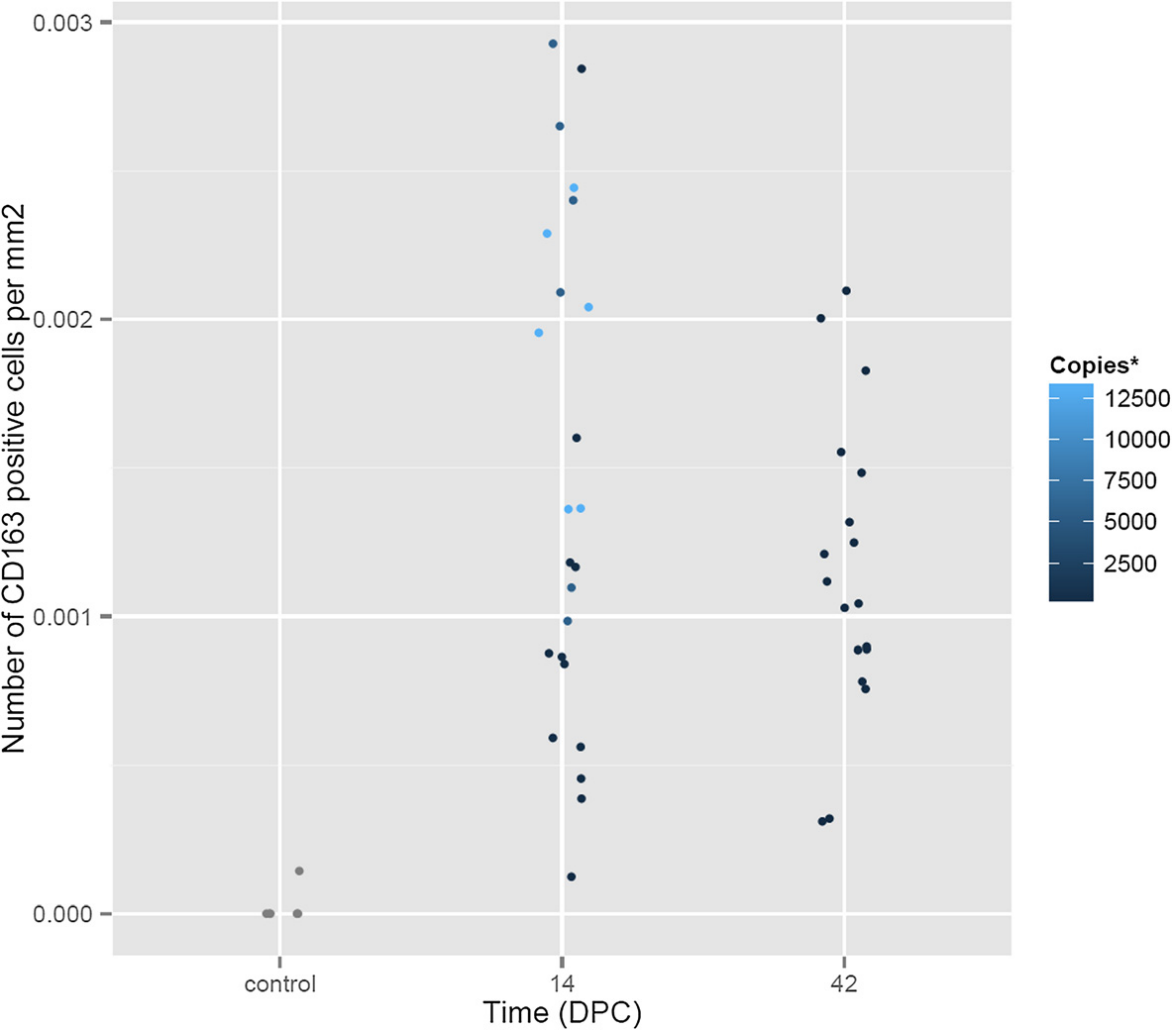
**Density of CD163 positive macrophages in pigs 14 and 42 days post challenge.** Comparison of the density of CD163 positive macrophages in the lamina propria of pig ileum infected with *L. intracellularis* 14 and 42 days post challenge (dpc). The colour gradations (*) denote the infection burden, measured using qPCR, as number of *L. intracellularis* 16S rRNA copies per nanogram of total genomic DNA.

Since an increase in intestinal macrophages has been previously associated with *L. intraellularis* infection, an immunohistochemical detection method was used to test if the CD163 transcript up-regulation was associated with a concomitant increase in macrophages specifically expressing the CD163 scavenger receptor protein [22]. At 14 dpc, there were moderate to large numbers of CD163 positive cells diffusely scattered throughout the lamina propria of the ileum in all four pigs (Figure 2A). At 42 dpc and in the one negative control pig ileum that was immunostained, only very small numbers of CD163 positive cells were present in the lamina propria (Figure 2B). Contrary to expectation, the difference in densities between the 14 dpc pigs and the 42 dpc pigs was not statistically significant (*p* = 0.48). However, there was a positive correlation between the density of CD163 positive cells and infection burden in the 14 dpc group (correlation = 0.79), measured as *L. intracellularis* 16S rRNA copies per nanogram of total genomic DNA (Figure 3).

## Discussion

Only three previous studies have focused on host gene expression in the context of PE [16,19,20]. The first of these studies mapped transcript changes over three time points using cultured mouse McCoy fibroblast-like cells as in vitro hosts [19]. This mesenchymal cell line is superior to others in its capacity to generate large numbers of *L. intracellularis* bacteria but extrapolation of its expression profile to the porcine crypt enterocyte (the natural target cell) may not be reliable [15,34,35]. The three time points were also assessed much earlier than the time of peak lesion development in experimentally infected pigs [21,36]. Nevertheless, that study provided valuable baseline data pertaining to early bacterial invasion and indicated that, even at this stage of infection, there was altered transcription of genes involved in cell cycle control.

The second study used intestinal tissues from field cases of pigs with diarrhoea to investigate cytokine expression, also using microarray methodology [20]. This provided whole animal information, something that cell culture models cannot, but the study was confounded by co-infection with porcine circovirus type 2 (PCV2) and, since diseased pigs were field cases, the findings were not correlated with stage of infection.

The most recent study introduced the use of laser capture microdissection in combination with RNA-seq analyses, allowing specific analysis of infected intestinal crypts from pigs experimentally challenged with a pure culture of *L. intracellularis* (strain PHE/MN1-00), negating any risk of confounding enteric infections. This novel approach facilitated the isolation of the cell of interest (crypt enterocyte) from the natural host, enabling a glimpse of the transcriptional alterations that may result from a combination of host and pathogen effects. Compared to that study, in which analysis was performed at a single time point coinciding with peak of infection, our own study examined multiple time points through initiation, peak and resolution of lesions. Specifically, we measured fold changes in gene expression for six weeks following challenge, allowing correlation with the previously described morphological lesions and level of infection in the same cohort of pigs [22]. In contrast to Vannucci et al. [16], we used a well-characterised comprehensive microarray platform which comprises over 47 000 probesets [28]. We also extended the interrogation to surrounding host tissues, specifically the intestinal lamina propria, where immune responses in particular are likely to be manifest.

Quantitative real time PCR analysis of *L. intracellularis*-specific genomic DNA detected peak infection at 14 dpc, correlating with previous reports [21,22,36]. There were some differences in the estimates of the infection burden between our qPCR data and the immunohistochemistry data reported by MacIntyre et al. [22]. Our qPCR assays detected infection in 80% of pigs tested between 21 and 35 dpc, whilst MacIntyre et al. [22] confirmed infection in only 25% of pigs tested at similar time points, using IHC. Since we tested tissues from the same cohort of pigs as MacIntyre et al. [22], the difference in detection rate cannot be due to differences in bacterial strain or source of pig. Rather, it is more likely that qPCR is more sensitive than IHC for detection of infection in frozen tissue. There is also accepted variation between PCR and immunologically based tests that further depends on whether tissue, faeces or blood are tested [37,38]. Different depths within the tissue block were also used for DNA extraction and IHC, which may have contributed to variability.

We found statistically significant changes in transcript levels for a total of 218 separate genes. The assumption that any cellular changes triggered by *L. intracellularis* are maximally expressed at time of peak infection burden (14 dpc in this study) was substantiated by fold changes that reached their maximum at this time point in most measured transcripts. At 14 dpc there were changes in 185 transcripts, 86% of which were down-regulated, a trend similar to that observed by Jacobson et al. [20]. This low number of altered transcripts is surprising, particularly as we used a highly comprehensive method that should have enhanced our ability to detect changes in gene expression [28]. One possible reason for this low detection level is individual host variation, intensified by the fact that the disease is unlikely to progress at exactly the same rate in different animals, as indicated in Figure 1, where there is variability in the accumulation of *L. intracellularis* 16S rRNA. The only way to overcome this using the same model would be to increase the number of pigs in each group, which is not realistic with a large animal model such as the pig. A more speculative reason is that the pathogen has a low impact on the host due to co-evolution or because it acts via a limited number of pathways that still have regulatory consequences for the infected cell. Further investigations of gene expression would help to determine whether this low level of transcriptional alteration and the predominance of down-regulated genes are genuine phenomena. Comparison of gene expression in intestinal tissues from pigs infected with a more aggressive pathogen, such as *Salmonella* or *Clostridium* spp., may also improve understanding in this regard.

We found the greatest fold down-regulation in *S100G* transcripts, the product of which is S100 calcium binding protein G (calbindin d9k). The precise role of S100G is uncertain but it facilitates intestinal absorption and transport of calcium under the influence of vitamin D [39]. None of the three previous analyses reported changes in this gene [16,19,20]. Down-regulation of various members of the solute carrier family (SLC) was also evident (Table 4). Solute carriers are membrane proteins responsible for the transport of nutrients and electrolytes. Those altered in our analysis are collectively responsible for the transport of oligopeptides, glucose, zinc, amino acids and neurotransmitters or the exchange of electrolytes, including Na^+^ and H^+^[40]. The expression of *CUBN*, which encodes a vitamin B12 transporter, was also down-regulated. While a few of these changes have been previously reported, our analysis expanded the list of SLC family member genes that are differentially expressed in infected pigs [16]. Vannucci et al. [16] detected down-regulation of *SLC7A3* and up-regulation of *SLC2A1*, findings that we were not able to confirm as these particular genes are not present on the Snowball microarray [28]. The down-regulation of genes controlling transport of nutritional elements could be due to failure of maturation of mucosal epithelial cells and may contribute to malabsorption diarrhoea, as previously suggested [16]. Our study found concomitant down-regulation of *SLC26A3*, a chloride/bicarbonate exchanger, and *SLC9A3*, the gene encoding NHE3. NHE3 is a sodium/hydrogen ion exchanger that is expressed by gastrointestinal epithelial cells and that facilitates absorption of sodium by enterocytes. Since these exchangers work in parallel and are essential for the regulation of intestinal water absorption, we postulate that, as a consequence of infection with *L. intracellularis*, their down-regulation leads to reduced sodium absorption and osmotic diarrhoea [41]. In our study several genes involved in regulating intestinal mucosal integrity, notably *MUC2* (encodes mucin-2), *RETNLB* (resistin-like beta), *TFF2* and *TFF3* (trefoil factors 2 and 3), and *CLDN15* (claudin 15) were down-regulated. Mucin-2 (the principal secretory mucin), resistin-like beta and the trefoil peptides are secreted by goblet cells [42,43]. TFF3 is important in epithelial restitution and cell migration, though not in proliferation, while mucin and TFF3 provide mucosal protection [43]. Resistin-like beta is a cytokine with multiple functions related to mucosal integrity, immunoregulation and intestinal glucose transport [44]. *CLDN15* (claudin 15) and *HEPACAM2* (hepacam family member 2) were the only tight junction-associated genes affected in our study. *HEPACAM2* is discussed later. Claudins are one of the four major integral membrane proteins but, pertinent to PE, in a homozygous claudin 15 deficient mouse model of mega-intestine, there is increased proliferation of crypt cells following weaning, suggesting a role for this protein in the modulation of crypt epithelial cell turnover [45]. In this study, *MUC2*, *TFF2*, *TFF3*, *RETNLB* and *CLDN15* expression levels were decreased between 3- and 9-fold at 14 dpc, phenomena that may lead to loss of mucosal integrity, disturbed cellular homeostasis and breakdown of intercellular relationships, particularly when considered in conjunction with expression changes in the SLC gene family and cubilin. As indicated above, presumably this reduced transcription is a further consequence of suppressed crypt epithelial cell maturation.

**Table 4:** **Comparison of solute carrier family gene expression changes between this study and Vannucci et al.**[16]

**Gene symbol**	**This study**	**Vannuci et al.**[16]	**solute carrier function**	**tissue-specific expression with BIOGPS**
SLC10A2	down-regulated	down-regulated	solute carrier family 10 (sodium/bile acid cotransporter family), member 2	n.d.**
SLC13A1	down-regulated	n.d	solute carrier family 13 (sodium/sulfate symporters), member 1	Ileum, colon
SLC15A1	down-regulated	n.d	solute carrier family 15 (oligopeptide transporter), member 1	intestine
SLC26A3	down-regulated	n.d	solute carrier family 26, member 3	intestine rectum
SLC30A10	down-regulated	n.d	solute carrier family 30, member 10	intestine
SLC31A1	down-regulated	down-regulated	solute carrier family 31 (copper transporters), member 1	several tissues
SLC5A1	down-regulated	down-regulated	solute carrier family 5 (sodium/glucose cotransporter), member 1	early intestine and bladder
SLC5A9	down-regulated	n.d	solute carrier family 5 (sodium/glucose cotransporter), member 9	n.d.**
SLC6A4	down-regulated	n.d	solute carrier family 6 (neurotransmitter transporter, serotonin), member 4	n.d.**
SLC7A9	down-regulated	n.d	solute carrier family 7 (cationic amino acid transporter, y + system), member 9	intestine
SLC7A3*	n.d.	down-regulated	solute carrier family 7 (amino acid transporter)	n.d.
SLC2A1*	n.d.	up-regulated	solute carrier family 2 (glucose tranporter)	n.d.

Note: nd = not detected.

*:not present on the Snowball microarray; **:expression not available on BioGPS.

The apparent ability of *L. intracellularis* to impede the maturation of crypt enterocytes is one of the most intriguing aspects of PE, more so because it is reversible [21]. However, underlying mechanisms of pathogenesis and resolution remain undefined. McOrist et al. [21] speculated that *L. intracellularis* could influence genes controlling differentiation and the first support for this hypothesis was provided by Oh et al. [19] who reported differential expression of several genes involved in cell cycle, cellular differentiation, apoptosis and signal transduction. Up-regulation of one such gene, *IGFBP3* (insulin-like growth factor binding protein 3), has been described by two separate and quite different PE studies [19,20]. In our study, cellular proliferation was one of the functional classes where transcriptional alterations were infrequent, with only one gene that is directly involved in cell cycle regulation, *CDK1*, up-regulated. CDK1 drives the cell through the G2 phase of the cell cycle, which immediately precedes mitosis [17]. A link between altered *CDK1* expression and PE has not been previously reported. Vannucci et al. [16] described up-regulation of CDK2-associated protein 1 (CDK2AP1), a growth suppressor that down-regulates CDK2 [46]. Since CDK2 has an effect on the cell cycle that is broadly similar to CDK1, it seems counter-intuitive that a CDK2 suppressor protein is up-regulated in PE. This merits further exploration. We detected 5-fold down-regulation in the transcription of *HEPACAM2*, a gene encoding hepacam which is an immunoglobulin-like cell adhesion molecule purported to be a tumour suppressor [47]. *HEPACAM* down-regulation has been reported in several human cancers and is believed to function by down-regulating c-myc and cyclin D1 [47-50]. Thus, reduced transcription of *HEPACAM2* associated with *L. intracellularis* infection could effectively “release the brakes” on c-myc and cyclin D1, leading to increased transcriptional activation and cellular proliferation. This is also an area worthy of deeper investigation.

In the context of local immunity, some of our findings are comparable with previous studies, such as the up-regulation of *SLA-3* (encodes MHCI), and the down-regulation of genes encoding elements of the CD3 T-cell receptor [16,22]. This apparent immunosuppression has been borne out by Jacobson et al. [20] who found limited expression of serum and tissue cytokines in natural cases of PE. Several previous studies have described macrophages in the lamina propria of infected pigs, often containing *L. intracellularis* organisms and sometimes peaking with maximum proliferation and burden of infection [10,22,50-53]. In our study, CD163 up-regulation at 14 dpc correlated with these previous results, although we could not convincingly confirm this immunohistochemically in tissues, possibly due to low pig numbers.

To conclude, this microarray-based study progresses the growing literature base that has more recently focused on the pathogenesis of PE, particularly at the molecular level. It provides evidence to support disruption of cell transport and mucosal integrity in pigs infected with *L. intracellularis*. Perhaps most interestingly, it has identified CDK1 and hepacam as potentially important molecules capable of influencing cellular proliferation in infected pigs.

## Competing interests

The authors declare they have no competing interests.

## Authors’ contributions

SHS, ADW, IVE contributed to the study design, acquisition, analysis of the data and manuscript preparation. IVM contributed to *L. intracellularis* quantification and analysis. NM contributed to the CD163 immunohistochemistry. ALA and TAA contributed to the study design, data analysis, supervision and manuscript preparation. All authors read and approved the final manuscript.

## Supplementary Material

Additional file 1 **Primer sequences for the microarray validation study.** Primer sequences used for the qPCR validation of the microarray data. Click here for file

Additional file 2 **List of transcripts regulated post infection with FDR < 0.1.** List of transcripts that were significantly regulated (FDR < 0.1) using Partek software, as described in Materials and methods Click here for file

Additional file 3 **Fold regulation of selected transcripts in ileum tissue during the experimental challenge of pigs with *Lawsonia intracellularis *** **LR189/5/83.** Transcript levels were measured using qPCR as described in Materials and methods and primer sequences described in Additional file 1. Fold regulation of transcript normalised over the untreated control and GAPDH mRNA transcript level as described previously [27]. 3 (*n* = 3), 7 (*n* = 4), 14 (*n* = 4), 21 (*n* = 4), 28 (*n* = 4), 35 (*n* = 2), 42 (*n* = 3) dpc. Click here for file

Additional file 4 **Gene ontology analysis of the data at 14 dpc using DAVID bioinformatics resources **[30]**.** The transcripts described in Additional file 2 were subjected to gene ontology analysis using the DAVID bioinformatics resources. Click here for file
