# Supplementary material for: Down-regulation of mechanisms involved in cell transport and maintenance of mucosal integrity in pigs infected with Lawsonia intracellularis
Source: Vet Res. 2014 May 20;45(1):55. doi: 10.1186/1297-9716-45-55 (PMC4031155; doi:10.1186/1297-9716-45-55)
Supplement: Additional file 1 — Primer sequences for the microarray validation study. Primer sequences used for the qPCR validation of the microarray data. [file 1297-9716-45-55-S1.docx]

| **Gene symbol** | **Ensembl**  **accession number** | **Primer sequence** |
| --- | --- | --- |
| **S100G** | ENSSSCT00000013286 | Fwd-AGGAGGAGCTGAAGCAACTG |
|  |  | Rev-TCCAGTTCCTGAAAGAGGTCA |
| **SLC7A9** | ENSSSCT00000027536 | Fwd-AGCACTGAACCCAAGACCAC |
|  |  | Rev-GTTGCTGAGCACAGACTTGG |
| **SLC6A4** | ENSSSCT00000019369 | Fwd-TTTCCTTCTCTCGGTCATCG |
|  |  | Rev-GCATCCATTTCGGTGGTACT |
| **SLC13A1** | ENSSSCT00000018083 | Fwd-ACAGCTTTGTTGCCTGGACT |
|  |  | Rev-ACTCCAATGAGCAGGAGGTG |
| **MUC2** | ENSSSCT00000014029 | Fwd-TCGAGTACATCCTGCTGACG |
|  |  | Rev-ACCGAGTCCTCTCTGTTCCA |
| **SLC30A10** | ENSSSCT00000011836 | Fwd-AAAAGTCCGAAGCCCTGAAT |
|  |  | Rev-TACACGGGTCCTCCTGTTTC |
| **SLC9A3** | ENSSSCT00000030360 | Fwd-TCACTCTACGGCGTCTTCCT |
|  |  | Rev-TGATGAAGAGCACCTCGTTG |
| **SLC5A9** | ENSSSCT00000004294 | Fwd-ATGACATTGCAGTGCTGGTC |
|  |  | Rev-CTCCCGGCCAGGAAGTAG |
| **TGFBR1** | ENSSSCT00000022448 | Fwd-GCGAGACAGGCCATTTGTAT |
|  |  | Rev-AAGCAGACTGGTCCAGCAAT |

**Additional file 1 Primers sequences for the microarray validation study.**
