# Supplementary material for: Down-regulation of mechanisms involved in cell transport and maintenance of mucosal integrity in pigs infected with Lawsonia intracellularis
Source: Vet Res. 2014 May 20;45(1):55. doi: 10.1186/1297-9716-45-55 (PMC4031155; doi:10.1186/1297-9716-45-55)
Supplement: Additional file 3 — Fold regulation of selected transcripts in ileum tissue during the experimental challenge of pigs with Lawsonia intracellularis LR189/5/83. Transcript levels were measured using qPCR as described in Materials and methods and primer sequences described in Additional file 1. Fold regulation of transcript normalised over the untreated control and GAPDH mRNA transcript level as described previously [27]. 3 (n = 3), 7 (n = 4), 14 (n = 4), 21 (n = 4), 28 (n = 4), 35 (n = 2), 42 (n = 3) dpc. [file 1297-9716-45-55-S3.pptx]

## Slide 1
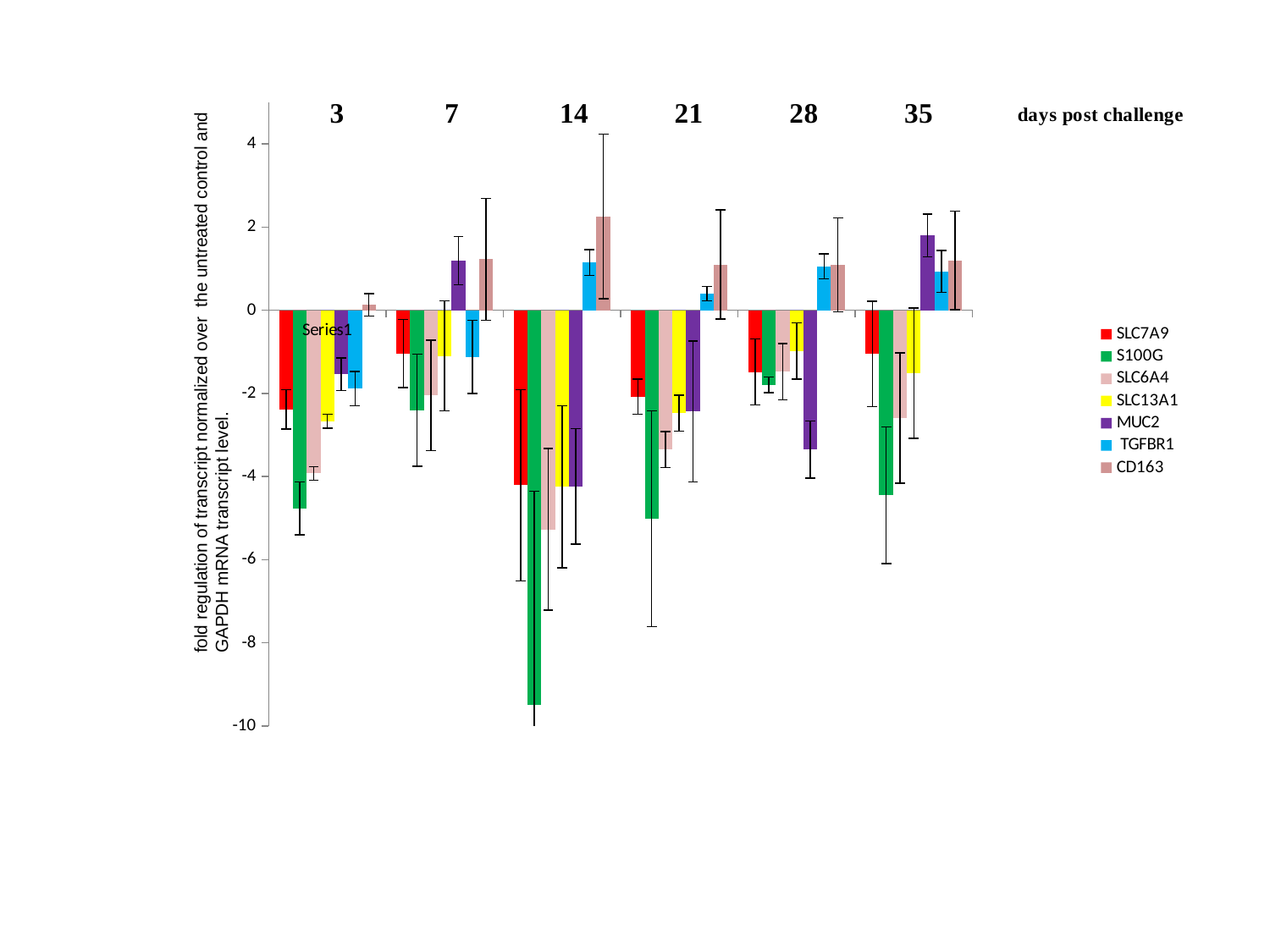

### Chart
| Category | SLC7A9 | S100G | SLC6A4 | SLC13A1 | MUC2 | TGFBR1 | CD163 |
|---|---|---|---|---|---|---|---|
| | -2.3844444444444406 | -4.76777777777778 | -3.92888888888888 | -2.6711111111111094 | -1.5411111111111115 | -1.8833333333333329 | 0.12888888888888891 |
| | -1.046666666666668 | -2.40666666666667 | -2.05277777777777 | -1.098888888888889 | 1.193333333333334 | -1.1207407407407384 | 1.2222222222222203 |
| | -4.209166666666666 | -9.493888888888893 | -5.27222222222221 | -4.248333333333335 | -4.23666666666667 | 1.144166666666667 | 2.258611111111108 |
| | -2.082222222222223 | -5.020000000000003 | -3.35333333333332 | -2.477777777777778 | -2.4350000000000023 | 0.39703703703703624 | 1.0955555555555527 |
| | -1.4866666666666681 | -1.7966666666666715 | -1.48333333333333 | -0.9766666666666713 | -3.3533333333333366 | 1.0522222222222162 | 1.093333333333329 |
| | -1.0533333333333346 | -4.45666666666667 | -2.59333333333332 | -1.515555555555558 | 1.7988888888888883 | 0.9303703703703713 | 1.1977777777777743 |
fold regulation of transcript normalized over the untreated control and
GAPDH mRNA transcript level.
